# Effects of follicular versus luteal phase-based strength training in young women

**DOI:** 10.1186/2193-1801-3-668

**Published:** 2014-11-11

**Authors:** Eunsook Sung, Ahreum Han, Timo Hinrichs, Matthias Vorgerd, Carmen Manchado, Petra Platen

**Affiliations:** Department of Sports Medicine and Sports Nutrition, Faculty of Sport Science, Ruhr-University Bochum, Gesundheitscampus Nord, Bochum, Haus 10, 44801 Germany; Department of Neurology, Ruhr-University Bochum, Kliniken Bergmannsheil, Bochum, Germany; Department of General and Specific Didactics, Faculty of Education, University of Alicante, Alicante, Spain; Department of Health and Fitness Management, Woosong University, Deajeon, South Korea; Swiss Paraplegic Research, Nottwil, Switzerland

**Keywords:** Eumenorrheic menstrual cycle, Isometric strength, Muscle fibre, Muscle diameter, Anabolic hormones

## Abstract

Hormonal variations during the menstrual cycle (MC) may influence trainability of strength. We investigated the effects of a follicular phase-based strength training (FT) on muscle strength, muscle volume and microscopic parameters, comparing it to a luteal phase-based training (LT).

Eumenorrheic women without oral contraception (OC) (N = 20, age: 25.9 ± 4.5 yr, height: 164.2 ± 5.5 cm, weight: 60.6 ± 7.8 kg) completed strength training on a leg press for three MC, and 9 of them participated in muscle biopsies. One leg had eight training sessions in the follicular phases (FP) and only two sessions in the luteal phases (LP) for follicular phase-based training (FT), while the other leg had eight training sessions in LP and only two sessions in FP for luteal phase-based training (LT). Estradiol (E2), progesterone (P4), total testosterone (T), free testosterone (free T) and DHEA-s were analysed once during FP (around day 11) and once during LP (around day 25). Maximum isometric force (F_max_), muscle diameter (Mdm), muscle fibre composition (No), fibre diameter (Fdm) and cell nuclei-to-fibre ratio (N/F) were analysed before and after the training intervention.

T and free T were higher in FP compared to LP prior to the training intervention (P < 0.05). The increase in F_max_ after FT was higher compared to LT (P <0.05). FT also showed a higher increase in Mdm than LT (P < 0.05). Moreover, we found significant increases in Fdm of fibre type ΙΙ and in N/F only after FT; however, there was no significant difference from LT. With regard to change in fibre composition, no differences were observed between FT and LT. FT showed a higher gain in muscle strength and muscle diameter than LT.

As a result, we recommend that eumenorrheic females without OC should base the periodization of their strength training on their individual MC.

## Introduction

In past decades, it has repeatedly been verified that serum concentrations of luteinizing hormone (LH), follicle-stimulation hormone (FSH), estradiol (E2) and progesterone (Prog) fluctuate during the menstrual cycle and that the level of androstenedione and testosterone reaches its peak prior to, or at the time of ovulation (Longcope 1986, Van Look and Baird 1980). This fluctuation of hormones during the menstrual cycle may influence 1) acute exercise performance during the respective phase, and 2) the trainability of muscle strength in a period when hormone milieu favours gain in muscle mass (Constantini et al. 2005; Janse de Jonge 2003; Lebrun 1994).

A number of studies on the effects of the menstrual cycle on exercise performance, however, show conflicting results. The above mentioned reviewers revealed in their newest available overviews in this topic that some studies showed a higher strength during the follicular phase than during the luteal phase whereas other studies reported the highest strength during the mid-luteal phase, while the majority of studies could not find any alteration in muscle strength over the menstrual cycle (Constantini et al. 2005; Janse de Jonge 2003; Lebrun 1994). In the recent 10 years only three studies on variation of muscle strength across the menstrual cycle that included hormone analysis for verification of the phase of the menstrual cycle when subjects were tested were found. None of them did find any effect of the phase of the menstrual cycle on isokinetic peak torque of knee extensors/flexors and maximum isometric strength of knee extension (Bambaeichi et al. 2004), on maximum voluntary isometric force of the first dorsal interosseus muscle (Elliott et al. 2003), or on handgrip strength and isokinetic muscle strength of knee extensors (peak torque), muscle endurance and one leg hop test (Fridén et al. 2003).

The effects of the phase of the menstrual cycle on trainability of strength in humans are even less clear, even though the available empirical evidence is promising. The only strength training intervention study using the different hormonal milieu of FP and LP as modulators of training adaptability analysed the possible divergent effects of training stimuli in either FP or LP on the amount of strength gain in healthy women (Reis et al. 1995). The authors described a higher trainability of strength of one-leg knee extensor muscles in 7 women when the respective leg was mainly trained for 4 weeks in FP compared to a training periodization without regarding the phase of the cycle. Despite the small number of subjects and wide inter-individual variability all subjects of this study showed higher strength adaptations during the follicular-phase based training.

One possible link between MC-depending training induced increases in muscle mass is the fluctuation of steroid hormones throughout the cycle and their possible effects on protein synthesis. The effects of estrogens on the human muscle have mainly been investigated in peri- and postmenopausal women. The striking decline in muscle strength occurring during the perimenopausal and postmenopausal period can be reversed by hormone replacement therapy (Bergström 1962; Jabbour et al. 2006). Discovery of 3 types of estrogen receptors has led to the finding that estrogen may govern the regulation of a number of downstream genes and molecular targets (Enns and Tiidus 2010; Lowe et al. 2010). Very recently estrogen receptor α and ß have been shown to be involved in muscle differentiation including slow myosin-heavy chain isoform (MHC) which is the dominant MHC isoform in type I fibres (Pellegrini et al. 2014) indicating that estrogens might influence muscle fibre type distribution. Further, one recent study reported that those women using hormone replacement therapy had significantly greater up-regulation of pro-anabolic gene expression both at rest and following eccentric exercise compared to a control group (Dieli-Conwright et al. 2009). Estrogens may also positively influence post-damage repair processes through activation and proliferation of satellite cells which are well known mechanism for skeletal muscle cell adaptation after a (strength) training stimulus (Enns and Tiidus 2010). Furthermore, it has recently been postulated that the beneficial effect of estrogens on muscle strength is accomplished by improving the intrinsic quality of skeletal muscle, whereby fibres are enabled to generate force, i.e. myosin strongly binds to actin during contraction, which might also lead to higher strength gains during training (Lowe et al. 2010).

Several recent clinical trials have indicated that testosterone supplementation at physiological doses in androgen-deficient women induce improvements in lean body mass clearly indicating the pro-anabolic effects of low-level testosterone on female skeletal muscle. These physiological effects may be critical for athletic performance and strength, albeit the effects of testosterone supplementation in women with serum androgen concentrations within the normal range has not been studied (Enea et al. 2011).

Only very few data exist on the physiological effects of progesterone (P4) on the female skeletal muscle cell. Recent studies have consistently found amino acid oxidation and protein degradation to be greater in the luteal phase (LP) compared with the follicular phase (FP) at rest and during exercise. It appears that P4 is responsible for the consistent finding of increased protein catabolism in LP, while estrogens may reduce protein catabolism (Oosthuyse and Bosch 2010).

All these studies support the hypothesis that estrogen and testosterone induce anabolic effects and progesterone has more katabolic effects on the skeletal muscle and that timing of strength training according to hormone concentrations might affect skeletal muscle adaptions. Indeed, Reis et al. (1995) did include analysis of steroid hormones in the above mentioned strength training intervention study. In short, they found estradiol in the training period being positively correlated with the muscle cross sectional area, estradiol before the training period being positively correlated with the development of maximal strength after one menstrual cycle, changes of progesterone between the luteal phases being negatively correlated with the development of maximal strength, and testosterone in the training period being positively correlated with the changes of the muscle cross sectional area. Although the authors summarised that the sample was very small and correlations do not necessarily represent cause-and-effect relationships, they concluded that the findings of their study suggest to consider not only testosterone and free testosterone but additionally the characteristic female hormones like estradiol and progesterone when investigating the interrelations between the physical performance and the endocrine system of female athletes.

Overall, the existing data indicate a more anabolic state in FP and the peri-ovulatory phase of the MC, compared to a more catabolic state in LP. No study, however, has so far combined the analysis of strength, macroscopic and cellular parameters after menstrual-cycle triggered training. Therefore the aim of the present study was to investigate the effects of menstrual cycle phase-based strength training on strength, macroscopic and microscopic muscle adaptations in a controlled training intervention study in healthy young females.

## Methods

### Subjects

20 healthy eumenorrheic women (mean (± SD) age: 25.9 ± 4.5 yr, height: 164.2 ± 5.5 cm, weight: 60.6 ± 7.8 kg) volunteered to participate in this study. Subjects were either untrained or moderately trained students from the university who partly participated in sport programs like aerobics or yoga. Some of them used a bicycle for transportation purposes. All subjects performed less than 2 hours of regular physical training per week. No one was experienced in or was currently performing resistance training. Subjects had not been taking oral contraceptives (OC) or any other hormonal treatments during the year prior to participation in this study and had no history of any endocrine disorders. All subjects had regular menstrual cycles (28.6 ± 2.3 days), and basal body temperature increased during the luteal phase of each cycle. Prior to the study, participants were informed about the purpose, procedures and risks of the study, however were not informed about the underlying hypotheses in order to avoid influence of motivation especially on maximal strength diagnosis. Blinding of training frequency concerning the phase of the menstrual cycle was not possible. Written informed consent was obtained from each participant. Approval for the study was obtained from the Ethics Committee of the Medical Faculty of Ruhr-University Bochum, Germany.

### Experimental design

This study was a controlled trial where the effects of two different menstrual cycle-based leg strength training programs were compared to each other (follicular phase-based- (FT) versus luteal phase-based training (LT)). Every participant performed both programs at the same time: one program with one leg, the other program with the other leg. To eliminate effects of a preference for one body side, the assignment of training programs to body sides was performed by randomization. The duration of the study for the individual participant was based on the length of the menstrual cycle. The entire study took five MC (two control cycles followed by three training cycles) which was equivalent to about 140 days considering that one menstrual cycle took approximately 28 days.

Throughout the whole study period the individual menstrual cycle integrity was analysed by measurements of basal body temperature and documented in a menstrual cycle calendar. If no clear increase in body temperature could be demonstrated around the middle of any of the menstrual cycles the subject was excluded from the study. Four subjects were excluded according to missing mid-cycle increase in body temperature. The remaining n = 20 subjects all had detectable increments of mid-cycle basal body temperature in each of five menstrual cycles. In the second control cycle and in each training cycle, blood samples for hormonal analysis were taken from a cubital vein on day 11 (late follicular phase) and on day 25 (late luteal phase) of the menstrual cycle when cycle length was between 27 and 29 days. When cycle length was between 30 and 31 days, venous blood was collected on day 13 and on day 27, and when cycle length was between 25 and 26 days, venous blood was collected on days 9 and 23. The condition of blood sampling was strictly standardized. Blood was taken between 8 and 9 am after an overnight fasting with the subjects staying in supine position. Strength of maximum isometric knee extension (F_max_) was measured around day 11 and around day 25 of the menstrual cycle, according to the individual length of the cycle. The sum of the diameters of rectus femoris, vastus intermedius and vastus lateralis muscle (Mdm) were measured on day 25 and muscle biopsies were taken from vastus lateralis muscle on day 27. Strength training was performed throughout the three consecutive training cycles. F_max_ was repeatedly measured throughout the training period on day 14 and on day 27 in each training cycle. In the third training cycle, the investigations which had been performed in the second control cycle (blood sampling, F_max_, Mdm, muscle biopsy) were repeated on the respective days.

We did not control for diet during the intervention period. Subjects, however, were instructed not to change their normal diet pattern and they did not reply any change when we asked them. Further, subjects were instructed to maintain their normal physical activity pattern outside the strength training. If they planned any one-leg training outside the study they were instructed not to do any of this training within the study period.

### Monitoring of menstrual cycle integrity

In order to determine the exact individual training and testing schedule, subjects measured their basal body temperature every morning throughout the entire study period at the same time before getting out of bed. The occurrence of ovulation was defined when an increase in basal body temperature of at least 0.3°C was measured (Kelly 2006; Owen 1975).

### Strength training program

The subjects completed three cycles of a controlled, supervised strength training program. The training was done four times a week: three times per week (Mondays, Wednesdays and Fridays) under supervision on a leg press machine and one time per week (Saturdays) at home with the subject’s own body weight (squats). Training participation was documented by the supervisors and additionally subjects documented their training participation in their training calendar. According to strong supervision, participation rate reached 92%. On the leg press subjects performed a one-leg sub-maximum strength training (80% of maximum strength of the respective leg) with three sets of 8–10 repetitions until exhaustion and with 3–5 minutes of recovery between sets. More weight was added for the leg press exercise when subject performed more than 12 repetitions. At home they performed three sets of 15–20 one-leg squats with 3–5 minutes of recovery between sets.

One leg was mainly trained in FP (FT) and the other leg mainly in LP (LT). The strength training program started individually with the 1st day of the menstrual cycle. As soon as basal body temperature increased more than 0.3°C for 3 days subjects changed their training leg. This scheme resulted in eight times training sessions in FP and just twice in LP in the follicular phase based training, and in eight times training sessions in LP and just twice in FP in the luteal phase based training session (Table 1). The respective two training loads in LP in the follicular phase based training (FT), and in FP in the luteal phase based training (LT) aimed at conserving the induced adaptation processes.

**Table 1:** **Study scheme and periodization of the strength training program of the follicular phase-based (FT) and the luteal phase-based training (LT) of the respective leg**

Pre training		3 training cycles						Post training
		1		2		3		
Hormones, Mdm, F_max_, muscle biopsy	Training program	FP	LP	FP	LP	FP	LP	Hormones, Mdm, muscle biopsy
	FT	xxxxxxxx	xx	xxxxxxxx	xx	xxxxxxxx	xx	
	LT	xx	xxxxxxxx	xx	xxxxxxxx	xx	xxxxxxxx	
		F_max_	F_max_	F_max_	F_max_	F_max_	F_max_	

FP: follicular phase, LP: luteal phase, FT: follicular phase-based training, LT: luteal phase-based training, Mdm: muscle diameter, F_max_: isometric maximal force of leg extension, x: one single training session.

### Hormone analysis

Venous blood was centrifuged after blood clotting and the serum kept frozen at −80°C until analysis. Each sample was analysed for estradiol (E2), P4, total testosterone (T) and free testosterone (free T), and dihydrotestosterone-sulfate (DHEA-s). E2, P4, T, and DHEA-s were assayed by immunochemistry (Elecsys® 1010 System, Roche Diagnostics GmbH), and free T was analysed by radioimmunoassay (Multi-Crystal LB 2111 gamma counter, Berthold Technologies GmbH & Co. KG).

The Elecsys 1010 analyser is a fully automatic, run-oriented analyser system for determination of immunological tests using the ECL/Origen electro-chemiluminescent process. ECL is a process in which highly reactive species are generated from stable precursors at the surface of an electrode. These highly reactive species react with one another producing light. The system measures samples in the form of serum and plasma. Depending on the test used, the results are produced either as quantitative or qualitative results. All components and reagents for routine analysis are integrated in or on the analyser. The measurement signals produced are used by the Elecsys 1010 to calculate the results. The measuring cell is a sealed chamber and consists of a working electrode, counter electrodes, a magnet and a photomultiplier. An immunological ECL test is made up of various pipetting steps, at least one incubation period and a measurement step. Generally, at least three test components (sample, reagent and microparticles) are pipetted into an assay cup. After the appropriate incubation period, the reaction mixture is aspirated into the measuring cell where the measurement process takes place. Each of these pipetting cycles is performed within a defined period (approximately 60 seconds).

The Multi Crystal LB 2111 gamma counter is a compact, robust and easy-to-use instrument for most applications where gamma-emitting isotopes are used. The 12 detectors are made of high-quality NaI well-type crystal providing best measurement geometry for gamma emitters located in the sample tubes. The principle of the analysis of free testosterone follows the basic principle of radioimmunoassay where there is competition between a radioactive and a non-radioactive antigen for a fixed number of antibody binding sites. The amount of I125-labelled testosterone analog bound to the antibody is inversely proportional to the concentration of the free testosterone present.

For the tests and reagents used in this investigation, the following lower detection limits were relevant: E2: 5.0 pg/mL; P4: 0.03 ng/mL; DHEA-s: 0.100 μg/dL; T: 2.5 ng/dL; free T: 0.18 pg/ml. Coefficients of variation were: E2: 4.4% (at 149 pg/ml); P4: 2.4% (at 1.57 ng/ml); DHEA-s: 2.8% (at 117 μg/dL); T: 10.2% (at 4.49 ng/dL); free T: 6.2% (at 1.87 pg/ml.). All blood samples from a single woman were analysed together to avoid inter-assay variations.

### Measurement of the strength of maximum isometric knee extension (F_max_)

F_max_ of the right and left leg was measured separately once in late FP (day 11) and once in late LP (day 25) in the 2nd control cycle and in each training cycle. F_max_ was determined on a leg press machine (Medizinische Sequenzgeräte, Compass, Germany) using a combined force and load cell (GSV-2ASD, ME-Messsysteme GmbH, Hennigsdorf, Germany). Prior to testing the subjects underwent a 10-min warm-up period of aerobic, low-resistance ergometer cycling and were familiarized with the test and the testing position (knee angle: 90°, ankle angle: 90°) on the leg press. Each measurement was repeated three times with 30 seconds of rest between the tests. The best result was selected for data analysis. Two subjects were not able to perform the isometric strength tests during the training cycles because of personal reasons. A reliability analysis was performed for the isometric measurement. The Intraclass Correlation Coefficient was 0.998, which indicates that the system has a high internal consistency (reliability).

### Determination of muscle diameter (Mdm)

Mdm of the rectus femoris, vastus intermedius and vastus lateralis muscle of the right and left leg was measured by real-time ultrasound imaging prior to and after training on day 25 in LP of the second control cycle and the third training cycle, analysing the distances between the outer and inner muscle fasciae using a Vivid I CE 0344 ultrasound device (GE Medical System, Solingen, Germany) with a parallel scanner (8 L-RS, 4.0–13.3 MHz) which provides 10 cm penetration depth of the sound wave and enables diagrams of deep lying muscles. The linear measurement of the shortest distance of the muscle depth was used. Previous studies showed that muscle cross-sectional area might reliably be measured using real-time ultrasound imaging (Martinson and Stokes 1991). Subjects prevented long-lasting static muscular tension for at least 30 minutes prior to the measurement in order to avoid alterations in Mdm (Reimer et al. 2004). All subjects lay supine with stretched legs on an examination couch without any pad, cushion or pillow underneath. Ultrasound images were obtained half-way between hip bone and knee cap and the transducer was placed gently on thighs to avoid compression and distortion of the underlying tissue. The transducer was held at an angle of 90° to the muscle borders to ensure a clear image. The images were displayed and frozen on the screen and they were photographed to measure the muscle diameter. The positions of transducer were recorded for each muscle to reproduce the exact position after training intervention. The means of three measurements of M. vastus medialis, M.vastus intermedius and M. vastus lateralis were taken at the same site for all subjects and the sum of three muscles diameters was calculated and used for data analysis (Mdm). Reliability analysis was performed for Mdm determination. The obtained ICC was 0.997, indicating a high reliability of the ultrasound imaging of Mdm used in this study.

### Histochemical analysis of muscle samples

Muscle samples (70 mg - 300 mg) were obtained from the vastus lateralis muscle of both right and left leg by the percutaneous needle biopsy technique (Bergström 1962). Nine subjects volunteered to participate in muscle needle biopsies on day 27 of the second control cycle and of the third training cycle (about two days after the last training). The muscle samples were removed from the needle and oriented cross-sectional, mounted in a Tissue-Tek OCT (Sakura Finetek Europe B.V., Zoeterwoude, Nederland) embedding medium, frozen in isopentane cooled by using liquid nitrogen, and stored at −80°C for subsequent histochemistry. Thin sections (10 μm) were cut in a cryostat at −20°C and mounted on cover glasses for staining.

Histochemical analysis for the determination of muscle fibre types (types Ι and ΙΙ) was performed with adenosine-triphosphatase (ATPase) staining procedures using an alkaline pre-incubation at pH 4.3 and 9.6 (Brooke and Kaiser 1970). Moreover, muscle cell nuclei were stained with hematoxylin and eosin for nuclei-to-fibre (N/F) ratio analysis (Yan 2000). Fibre type counting and measurements were performed on photographs. For muscle fibre composition, an average of 288 fibres from each sample was identified, and the percentage of each fibre type was calculated. For the muscle fibre diameter averages of 62 fibres (range 20–119) from each fibre type (type Ι and type ΙΙ) were selected and measured using Cell-D life science documentation software (Olympus life and material science Europe GmbH, Hamburg, Germany).

### Statistical analysis

Data are presented as mean values with SD. Normality of distributions was proved by the Kolmogorov-Smirnov test. A two-tailed paired t-test was used to evaluate improvements in training workload, of F_max_, Mdm, fibre composition, fibre diameter and N/F between values before (pre) and after the training intervention (post). ANOVA with repeated measures was used to determine main effects of time, cycle phase, and time by cycle phase interaction. In all cases, P values <0.05 (two-tailed paired t-test) were taken to indicate statistical significance. The intra-class correlation coefficient of repeated measurements (ICC) was determined to evaluate reliability of the determination of F_max_ and Mdm (McGraw and Wong 1996, 2004). Effect size and statistical power for changes in F_max_, Fdm, and alterations in muscle morphological characteristics were analysed post hoc as a function of significance level α, the respective sample sizes, and the means and standard deviations of differences using G*Power power analysis program (Faul et al. 2007, Faul et al. 2009, Cohen 1988).

## Results

### Number of training sessions

The total number of single-leg training sessions was approximately 28 sessions per leg and was not different between FT and LT (FT: N = 28.6 ± 1.7; LT: N = 28.1 ± 1.9; P > 0.05).

### Hormone concentrations

We did not find any significant differences in the serum concentrations of E2 and DHEA-s between day 11 and day 25 of the menstrual cycle prior to training, while P4 was significantly higher (effect size: 0.995, power: 0.98), and T and free T were significantly lower on day 25 compared to day 11 (effect sizes: 0.67 and 0.81, power: 0.76, 0.88, respectively) (Table 2). After the strength training period, E2 became significantly higher in LP compared to FP (effect size: 0.56, power: 0.61), and P4 became higher in LP compared to LP prior to the training intervention (effect size: 0.67, power: 0.77), while DHEA-s remained unchanged. The differences in T and free T between both days were no longer detectable after the training period. T declined significantly from pre- to post-training in FO (effect size: 0.64, power: 0.73), and free T tended to decline from pre- to post-training in FO (p = 0.066, effect size: 0.54, power: 0.56).

**Table 2:** **Serum concentrations of E2, P4, DHEA-s, T and free T in the follicular phase (FP, around day 11) and the luteal phase (LP, around day 25) before and after three months of follicular phase-based or luteal phase-based strength training (N = 20)**

	Pre-training		Post-training	
	FP	LP	FP	LP
E2 (pg/ml)	124 ± 104	114 ± 71	92 ± 70	142 ± 41†
P4 (ng/ml)	0.82 ± 0.53	5.66 ± 3.93†	0.78 ± 0.50	8.36 ± 3.33†*
DHEA-s (μg/ml)	2.65 ± 1.13	2.52 ± 0.83	2.55 ± 0.73	2.58 ± 0.73
T (ng/ml)	0.44 ± 0.20	0.35 ± 0.18†	0.37 ± 0.14*	0.37 ± 0.15
Free T (pg/ml)	2.57 ± 0.86	1.94 ± 0.62†	2.14 ± 0.62*	2.06 ± 0.60

E2: estradiol, P4: progesterone, T: testosterone, pre/post-training: before/after three months of strength training.

FP: follicular phase, LP luteal phase, *: P <0.05 post training vs. pre training, †: P <0.05 LP vs. FP.

### Maximum isometric muscle strength

**Figure 1:**
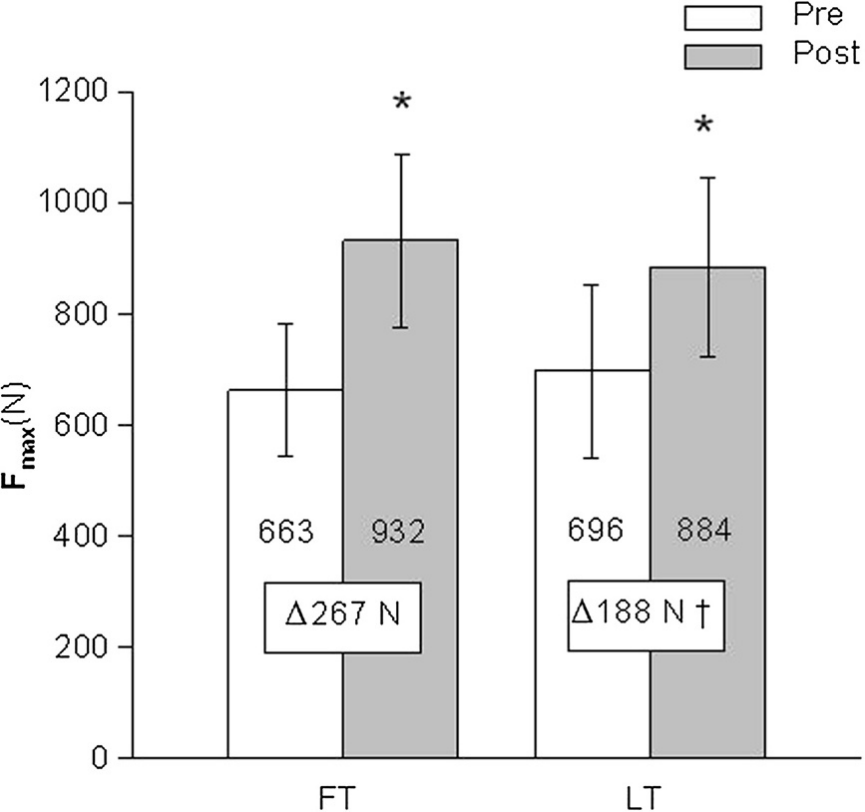
Fmax before and after three months of follicular phase-based (FT) or luteal phase-based (LT) strength training (N=20); Pre: before training, Post: after training, *: P < 0.05 post training vs. pre training, †: P < 0.05 FT vs. LT.

**Figure 2:**
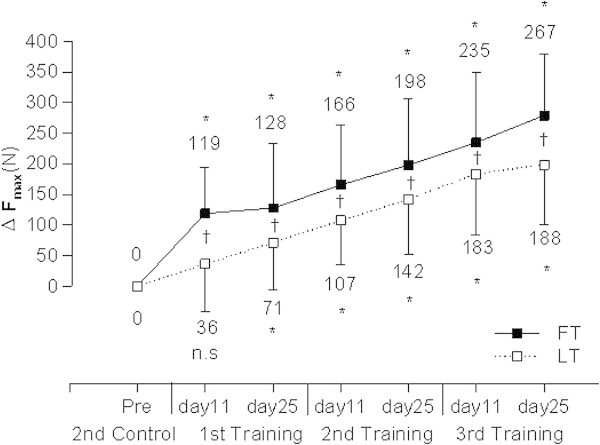
Increase in Fmax compared to the pre-training value during follicular phase-based (FT) or luteal phase-based (LT) strength training (N=18); Pre: before training, Control: control cycle, Training: training cycle, Day 11: analysis around day 11; Day 25: analysis around day 25; n.s.: not significant; *: P < 0.05 compared to pre training, †: P < 0.05 FT vs. LT.

F_max_ of one-leg knee extension muscles did not differ between FO and LU prior to the training period. F_max_ of knee extension muscles increased significantly (P < 0.05) after both types of training periodization compared to the pre-training level (Figure 1). Absolute increase in F_max_ was significantly smaller after LT (∆LT: 188 ± 98 N) compared to FT (∆FT: 267 ± 101 N) (P < 0.05, effect size: 0.87, power: 0.96). F_max_ increased progressively during FT and LT compared to the mean of both measurements in the control cycle (Figure 2).

### Muscle diameter

**Figure 3:**
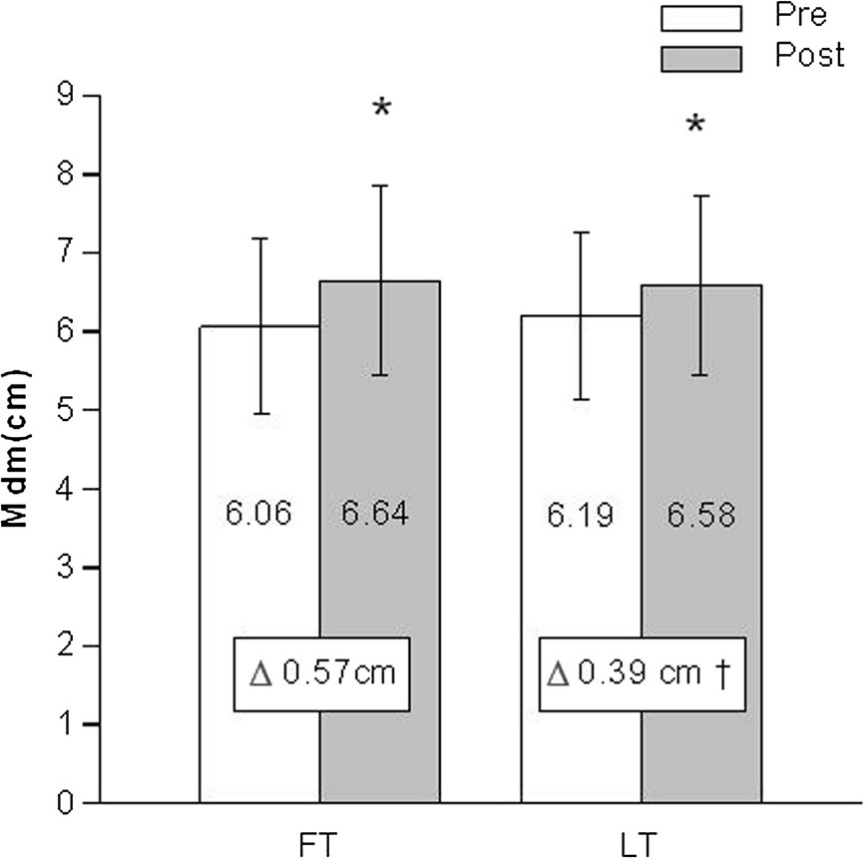
Sum of the diameters of rectus femoris, vastus intermedius and vastus lateralis muscle before and after 3 months of follicular phase-based (FT) or luteal phase-based (LT) strength training (N=20); Pre: before training, Post: after training, *: P < 0.025 post training vs. pre training, †: P < 0.025 FT vs. LT.

The sum of Mdm of the three muscles increased significantly (P < 0.05) after both types of training periodization compared to the pre-training level. Increase in Mdm was significantly higher after FT (∆FT: 0.57 ± 0.54 cm) compared to LT (∆LT: 0.39 ± 0.38 cm) (P < 0.05, effect size: 0.47, power: 0.52, Figure 3).

### Muscle fibre characteristics

Fibre type distribution remained nearly the same after both kinds of strength training periodization with about 40% type I fibres and 60% type II fibres (Table 3). Fdm increased significantly after FT in type II fibres (P < 0.05, effect size: 0.94, power: 0.70) and tended to increase after LT in type II fibres (P = 0.095, effect size: 0.63, power: 0.38), but remained the same in type I fibres after FT and LT. The N/F ratio increased significantly after FT (P < 0.05, effect size: 0.90, power: 0.66) and remained unchanged after LT.

**Table 3:** **Muscle fiber type distribution (No), fiber diameter (Fdm) and nuclei-to-fiber ratio (N/F) before and after three months of follicular phase-based or luteal phase-based strength training (N = 9)**

	Pre-training				Post-training			
	FT		LT		FT		LT	
	Type Ι	Type ΙΙ	Type Ι	Type ΙΙ	Type Ι	Type ΙΙ	Type Ι	Type ΙΙ
No (%)	40.9 ± 9.1	59.1 ± 9.1	41.8 ± 13.6	58.2 ± 13.6	40.3 ± 11.1	59.7 ± 11.1	40.5 ± 13.0	59.5 ± 13.0
Fdm (μm)	54.5 ± 5.1	45.8 ± 5.8	54.0 ± 7.4	46.8 ± 7.9	56.7 ± 7.1	52.5 ± 7.0*	57.0 ± 3.4	51.9 ± 7.3#
N/F	2.9 ± 0.4		3.4 ± 0.8		3.8 ± 1.1*		3.4 ± 0.7	

FT: follicular phase-based training, LT: luteal phase-based training, *: P <0.025 post-training versus pre-training, #: P = 0.045 post-training versus pre-training.

## Discussion

The most important finding of our study is a significantly higher (power 0.96) increase in F_max_ after FT compared to LT (Figure 1). This is in line with the main finding of (Reis et al. 1995) who described a higher percent increase in F_max_ after the second training cycle in the follicular phase-trained leg compared to the regularly trained leg (33% increase vs. 13% increase in F_max_).

The second important finding of our study is a significantly higher (power 0.52) increase in Mdm after FT compared to LT, which is in line with the higher increase in F_max_ after FT. This higher increase in Mdm after FT may be explained by a higher ratio between protein synthesis and protein breakdown during or after each strength training session in FP compared to LP (Oosthuyse and Bosch 2010).

This study is the second one to address the planning of strength training with respect to hormonal fluctuations during the MC and the first to include the analysis of muscle cell parameters. We analysed the effects of a longer-lasting training period and we clearly varied the strength training periodization between FP and LP in contrast to the first study (Reis et al. 1995) which strengthens the view that menstrual cycle induced alterations in hormone concentration are one probable cause for the differences in the height of strength increase. Plasma hormone concentrations, however, represent a balance among production, metabolism, utilization, clearance, and plasma volume. With our measurements we only considered one variable of a complex system, which additionally represents just the actual concentration. To minimize the variations in this system we strictly standardized the conditions during blood sampling, although this remains a limitation of the study.

The more pronounced increase in muscle strength and muscle diameter in FT compared to LT could be explained by the higher concentrations of T and free T during FP compared to LP in the pre-training and probably also first training period (Table 2), although power of the effects is not very high and we analysed hormone concentration rather in the late follicular than in the early follicular phase which makes interpretation of the hormone values somewhat weak. This is another limitation of the present investigation. Data in the literature on variation of anabolic hormones throughout the menstrual cycle, however, are consistent with our most relevant findings. Since androgen secretion from the ovary is under luteinizing hormone (LH) control, it is not unexpected that ovarian androgen secretion varies through the cycle: the blood levels of T have been described as lowest in the early follicular phase and then rising to their highest levels just prior to, or at the time of ovulation and then gradually fall during the luteal phase (Alexander et al. 1990; Jabbour et al. 2006; Longcope 1986). Therefore, it seems probable that they account for differences in strength, muscle diameter, and muscle cell characteristics between follicular- compared to luteal phase-based strength training.

Furthermore, the higher increases in F_max_ and Mdm after FT might also be explained by menstrual cycle-dependent alterations of estradiol and progesterone. It has long been demonstrated that the ovarian hormones fluctuate during the menstrual cycle (Oosthuyse and Bosch 2010; Reilly 2000). E2 peaks prior to ovulation and during LP, while P4 reaches its highest values during LP after ovulation (Van Look and Baird 1980). The ovarian hormones are known to have a noticeable influence on protein metabolism at rest and during exercise. It appears that progesterone is responsible for the consistent finding of increased protein catabolism in LP, while estrogen might reduce protein catabolism (Oosthuyse and Bosch 2010).

The similar concentrations in E2 around day 11 and day 25 prior to the training period in our study are probably due to the fact that day 11 represents a phase very close to ovulation, when E2 is already elevated compared to early and middle FP (Van Look and Baird 1980). A time point during the early follicular phase (around day 4 to 6) of the menstrual cycle probably would have led to clearly lower concentrations in FP compared to LP for these hormones. This is a limitation of the study.

The higher value of E2 in LP compared to FP, the increase in P4 in LP, and the decline in T and free T in FP after the strength training intervention may be due to exercise- and training-induced changes in menstrual cycle physiology, including alterations in feedback regulation of steroid hormones. Recently, serum estradiol and progesterone have been shown to increase in the mid-luteal phase, and testosterone has been shown to decrease in the early follicular and in the mid-luteal phase after a single bout of resistance exercise in healthy young women, indicating that the responses of steroid hormones to acute resistance exercise are different between hormones and vary between menstrual cycle phases in young women (Nakamura et al. 2011). The authors concluded that the menstrual cycle state may influence the exercise training-induced skeletal muscular adaptation, and that it would be possible for training programs for eumenorrheic women to be timed in accordance with the menstrual cycle in order to maximize anabolic effects.

We found an increase in serum estradiol and P4 in LP after 3 months of strength training, suggesting that the acute strength training-induced increase in the luteal phase described by Nakamura et al. (2011) might chronically lead to an increase in serum estradiol and progesterone basal concentration in this cycle phase. Further, we found a decline in serum testosterone in FP after 3 months strength training, suggesting that the acute strength training-induced decline described by Nakamura et al. (2011) might chronically lead to a reduction in serum testosterone basal concentration in this phase.

A remarkable finding of the muscle biopsy analysis was the significant increase in the type II fibre diameter after FT compared to only a tendency for an increase after LT. Resistance training has been shown to increase the volumes of myofibrils, of the interfibrillar space, of mitochondria and lipid droplets in females (Wang et al. 1993). An increase in the number and or size of myofibrils requires an increase in specific protein biosynthesis, which is dependent on anabolic agents such as testosterone and estrogens. Therefore, the slightly higher increase in cell diameter of type II fibres after FT compared to LT in our study is again in line with the higher increase in muscle strength and muscle diameter after FT compared to LT, and the well-known menstrual cycle-dependent alterations in anabolic hormones.

Interestingly, the N/F ratio increased after FT but remained unaffected after LT. Enlargement of muscle fibres is accompanied by an increase in the myonuclear number. Existing myonuclei are able to support a certain level of fibre hypertrophy. However, when the transcriptional activity of existing myonuclei reaches its maximum, the enhancement of the number of myonuclei is thought to become involved in the enhancement of protein synthesis (Kadi 2008). A substantial increase in the size of myofibres in the muscles requires the availability of satellite cells that can provide additional myonuclei to support hypertrophy (Adams 2006). A variety of alterations in the surrounding environment of the satellite cell, including mechanical and growth factors, as well as hormonal signalling including testosterone could regulate the activation and proliferation of satellite cells (Kadi 2008). Furthermore, sex-mediated differences in muscle-fibre regeneration and satellite-cell numbers may be directly attributed to estrogenic influence, and estrogen may exert its influence on post-exercise muscle-satellite cell populations through events upstream of satellite-cell activation (Enns and Tiidus 2010). Taken together, although spoken with caution to not over-interpret data, our results underpin a possible role of hormonal alterations, both of testosterone and estrogens, throughout the menstrual cycle in the process of satellite-cell incorporation-induced muscle hypertrophy.

## Conclusions

In conclusion, this study demonstrated that follicular phase-based strength training induced a greater effect on muscle strength, muscle and type II fibre diameters, and nuclei-to-fibre ratio compared to luteal phase-based strength training in untrained and moderately trained women. We recommend that eumenorrheic females without oral contraception base the periodization of strength training on their individual menstrual cycle.

## Abbreviations

ATPase: Adenosine triphosphase
DHEA-s: Dehydroepiandrosterone sulfate
E2: Beta-estradiol
Fdm: Fibre diameter
Fmax: Maximum isometric force
FP: Follicular phase
Free T: Free testosterone
FT: Follicular phase-based training
LP: Luteal phase
LT: Luteal phase-based training
MC: Menstrual cycle
Mdm: Muscle diameter
N/F: Cell nuclei-to-fibre ratio
No: Muscle fibre composition
OC: Oral contraception
P4: Progesterone
T: Total testosterone.
